# Interleukin-1 and Systemic Sclerosis: Getting to the Heart of Cardiac Involvement

**DOI:** 10.3389/fimmu.2021.653950

**Published:** 2021-03-23

**Authors:** Giacomo De Luca, Giulio Cavalli, Corrado Campochiaro, Cosimo Bruni, Alessandro Tomelleri, Lorenzo Dagna, Marco Matucci-Cerinic

**Affiliations:** ^1^Unit of Immunology, Rheumatology, Allergy and Rare diseases (UnIRAR), IRCCS San Raffaele Hospital, Milan, Italy; ^2^Vita-Salute San Raffaele University, Milan, Italy; ^3^Department of Experimental and Clinical Medicine, University of Florence, and Division of Rheumatology AOUC, Florence, Italy

**Keywords:** systemic sclerosis (scleroderma), heart inflammation, interleukin-1, inflammasome, cellular metabolism

## Abstract

Systemic sclerosis (SSc) is rare, severe connective tissue disease characterized by endothelial and vascular damage, immune activation, and resulting in inflammation and fibrosis of skin and internal organs, including the heart. SSc is associated with high morbidity and mortality. Cardiac involvement is frequent in SSc patients, even though often asymptomatic at early stages, and represents one of the major causes of SSc-related mortality. Heart involvement has a variable clinical presentation, and its pathogenesis is not completely understood. Myocardial fibrosis is traditionally considered the immunopathologic hallmark of heart involvement in SSc. This unique histological feature is paralleled by distinctive clinical and prognostic features. The so-called “vascular hypothesis” represents the most credited hypothesis to explain myocardial fibrosis. More recently, the prominent role of an inflammatory myocardial process has been identified as a cardinal event in the evolution to fibrosis, thus also delineating an “inflammation-driven pathway to fibrosis”. The pro-inflammatory cytokine interleukin (IL)-1 has an apical and cardinal role in the myocardial inflammatory cascade and in cardiac dysfunction. The primary aim of this perspective article is: to present the emerging evidence on the role of IL-1 and inflammasome in both SSc and heart inflammation, to review the complex interplay between cellular metabolism and inflammasome activation, and to discuss the rationale for targeted inhibition of IL-1 for the treatment of SSc-heart involvement, providing preliminary experimental and clinical data to support this hypothesis.

## Introduction

Heart involvement is frequent and is a major cause of mortality in systemic sclerosis (SSc), being responsible for up to 30% of disease-related deaths (1–3). Heart involvement has a variable clinical presentation: at early stages most patients are asymptomatic, but some go on to develop arrhythmias, dyspnea, chest pain, and heart failure (HF) (1, 4–9). In comparison to other inflammatory myocardial disease, myocardial fibrosis is usually considered the immunopathologic hallmark of SSc heart disease. This unique histological feature is paralleled by distinctive clinical and prognostic features (10, 11).

The most credited hypothesis to explain myocardial fibrosis is the one attributed to “vascular” (12): intermittent vascular spasm, ischemic necrosis, and reperfusion injury are considered pivotal mechanisms in fibrogenesis. More recently, the prominent role of an inflammatory myocardial process, clinically identified as a myocarditis, has been also identified as a crucial event in the evolution to fibrosis, thus also delineating an “inflammation-driven pathway to fibrosis” (1, 4, 13–18). In this “bimodal” ischemic-inflammatory pathogenic model, reperfusion products and pro-inflammatory cytokines may jointly orchestrate SSc-related heart involvement (SSc-HI). Therefore, the fact that only the inflammatory pathway to fibrosis is similar to other inflammatory cardiomyopathies (HF, dilated cardiomyopathy [DCM], virus-negative myocarditis) makes myocardial involvement in SSc a really peculiar and complex multifaceted event (19–24).

The pro-inflammatory cytokine interleukin (IL)-1 has an apical and cardinal role in the myocardial inflammatory cascade and in cardiac dysfunction (24). In this article, we provide an expert perspective on the emerging evidence on the role of IL-1 in both SSc and heart inflammation, and discuss the rationale for targeted inhibition of this cytokine for the treatment of SSc-HI.

## IL-1 Family and IL-1 Biology

The IL-1 cytokines family includes seven members with agonistic activity (IL-1α, IL-1β, IL-18, IL-33, IL-36α, IL-36β, and IL-36γ) and four members with antagonistic functions (IL-1Ra, IL-36Rα, IL-37, and IL-38) (25–28).

IL-1 is an archetypal pro-inflammatory cytokine. The term IL-1 hints at two different molecules, IL-1α and IL-1β, which share a significant sequence homology and bind the same IL-1 type-I receptor (IL-1RI), which then transduces pro-inflammatory signals and leads to the synthesis and expression of myriad secondary inflammatory mediators (29). IL-1α is constitutively present in epithelial cells as a fully active pro-inflammatory mediator, and is released upon cell death thus acting as an “alarmin”. Alarmins are a group of intracellular mediators, of which High Mobility Group Box 1 (HMGB1) likely represent the best characterized member, signaling tissue damage and activating inflammatory patrolling when found in the extracellular space. In scleroderma, mechanisms leading to alarmin release include ischemic cell death or inflammation-mediated tissue damage (30). Conversely, IL-1β is primarily produced by myeloid cells as an inactive precursor. Production of the mature pro-inflammatory cytokine follows activating cleavage of the precursor by an intracellular molecular complexes termed “inflammasomes” (31). To dampen excessive inflammation, the same cells that produce IL-1α or IL-1β also synthesize diverse regulatory molecules, including the IL-1 receptor antagonist (IL-1Ra). IL-1 signaling and IL-1-mediated inflammation are prevented by competitive binding of IL-1Ra to IL-1RI (30–33), thus curbing IL-1-mediated inflammation.

## IL-1 and the Inflammasome in SSc

Expression or biologic activity of most IL-1 family cytokines can be abnormal in many autoimmune diseases, including SSc (34). IL-1α regulates differentiation of fibroblast into myofibroblast, as well as myofibroblast longevity, which are considered central events in SSc (34). Indeed, dermal fibroblasts from SSc patients cultured *ex vivo* express higher levels of intracellular IL-1α than healthy counterparts (35). Immunohistochemical studies indicated that intracellular IL-1α is also markedly expressed in fibroblasts isolated from skin lesions of SSc patients; in addition, endogenous IL-1α induces fibroblast proliferation and production of collagen by inducing IL-6 and platelet-derived growth factor (PDGF) (36). Consistently, the production of IL-6, suppression of IL-1α through IL-1α siRNA results in decreased PDGF and procollagen production in SSc-affected fibroblasts (37), whereas overexpression of IL-1α through transfection in healthy fibroblasts promotes differentiation into a SSc-related phenotype (34). In SSc fibroblasts, the NLRP3 inflammasome is over-expressed and caspase-1 activity is up-regulated with consequent increased production of IL-1β and IL-18, whereas inhibition caspase-1 and inflammasome activity abrogated the myofibroblast phenotype in SSc dermal and lung fibroblasts (38–40). A separate study revealed that SSc fibroblasts exhibit increased synthesis of micro-RNA (miR)-155, which can also be induced by IL-1β (22). To date, miR-155 was implicated in various biological processes, including inflammation, immunity, and fibrosis (41). MiR-155, moreover, has been involved in cardiac remodeling, and miR155 deletion or inhibition reduced inflammatory and fibrotic responses in animal models of cardiac fibrosis induced by angiotensin-II (Ang-II) or diabetes (42, 43). In addition, miR-155 is required for the synthesis of collagen induced by activation of the inflammasome. Indeed, inhibition of caspase-1 activity abrogated miR-155 expression and significantly dampened collagen synthesis in a bleomycin-induced SSc mouse model (22).

In SSc patients, high levels of IL-1βcan be observed both in the bronchoalveolar lavage fluid (BAL) and in the serum (44). In the affected skin of SSc patients, IL-1β and IL-18 were significantly over-expressed, a finding correlating with the area of skin fibrosis assessed by the modified Rodnan skin score (mRSS) (38). This finding is not surprising, since IL-1β also induces myofibroblast activation, endothelial to mesenchymal transition, and fibrosis through IL-6 and TGF-1β (45).

IL-1α is an intracellular cytokine which is rarely if ever detectable in the circulation, including in SSc patients (35). Similarly, even though associations between genes encoding IL-1 family cytokines and SSc susceptibility were revealed by genome-wide association studies, the results are not conclusive (46–51).

## Inflammasome, IL-1, and Metabolism

Recent studies have identified a strong interplay between cellular metabolism and inflammasome activation (52). Specifically, NLRP3 inflammasome is regulated by cellular metabolism, and growing evidences suggest that cellular metabolism is a crucial driver for macrophage polarization and inflammation, as well as myofibroblast differentiation and fibrosis (53, 54).

**Figure 1:**
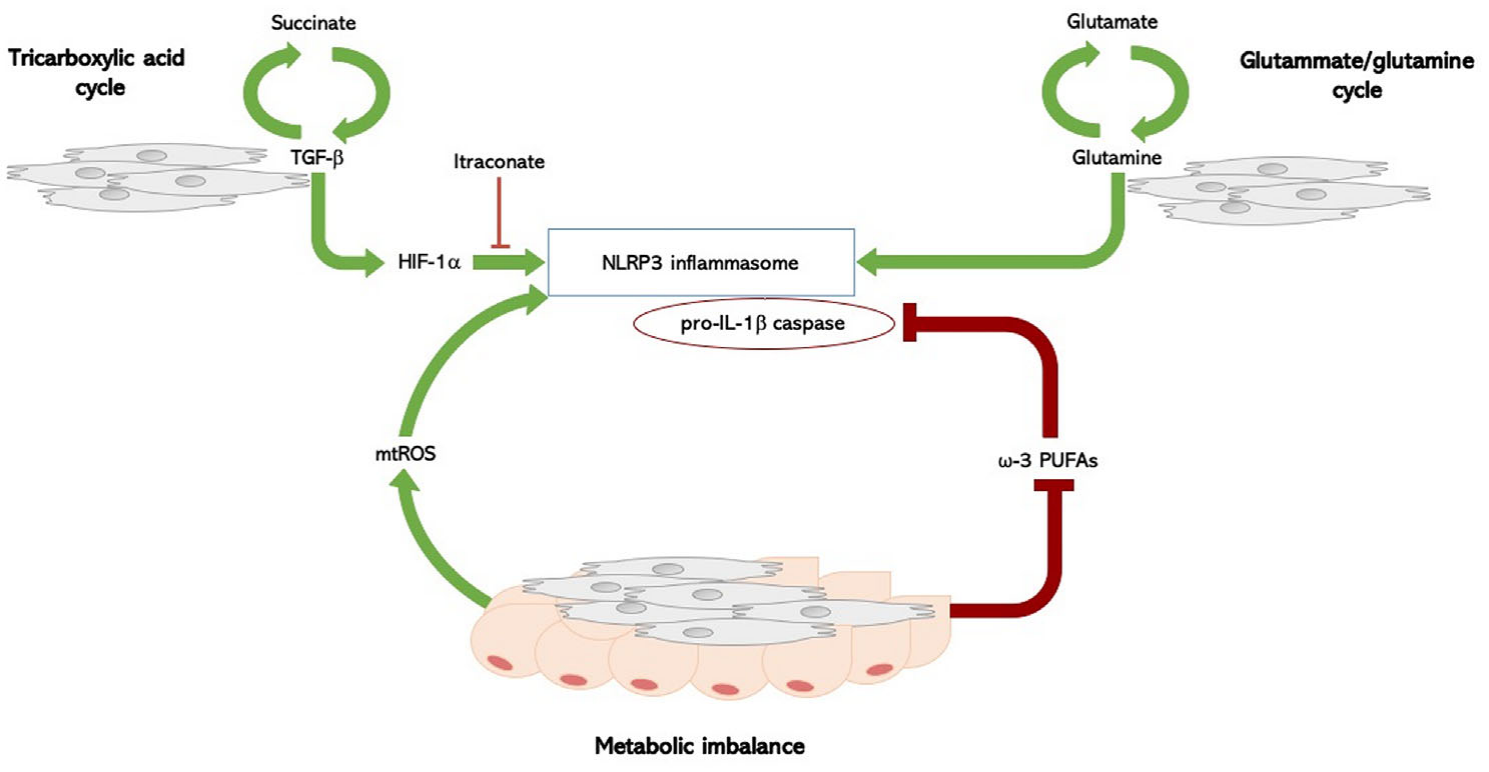
Inflammasome and metabolism in systemic sclerosis. Molecular pathways involved in the metabolic regulation of the NLRP3 inflammasome in SSc: TCA cycle, fatty acid imbalance, and amino-acid metabolism. In SSc, a complex biological loop in which the TGF-β1 rich microenvironment, the upregulated glutamine metabolism, and the fatty acid dysregulation, could lead to both inflammasome activation with IL-1β release and myofibroblasts differentiation, thus foraging the inflammation-driven fibrosis. Succinate is formed in the TCA cycle; its levels increase the TGF-β1-induced HIF-1α expression, promoting fibroblast differentiation. High levels of succinate can support IL-1β expression by stabilizing HIF-1α for IL-1β transcript expression to occur. This process is inhibited by itaconate. TGF‐β1 itself is able to enhance succinate production, thus foraging this biological loop. SSc fibroblasts have an increased glutaminase expression, and an altered glutamine metabolism is an ubiquitous trait in SSc. The glutammate-glutamine pathway activates the NLPR3 inflammasome. Fatty acid metabolism has been implicated in the regulation of NLRP3 inflammasome: metabolic imbalance itself act as a cue to activate an inflammatory response, though the production of mitochondrial reactive oxygen species (mtROS), which directly activate the NLRP3 inflammasome. PUFAs, particularly ω-3 PUFA, regulate NLRP3 inflammasome activation, acting as potent inhibitors of both caspase-1 activation and IL-1β release. Fatty acid metabolism is dysregulated in SSc, and intradermal adipose tissue is atrophied and replaced by collagen-rich fibrous tissue in SSc. SSc, systemic sclerosis; TGF-β1, transforming-growth factor beta-1; HIF-1α, hypoxia-inducible factor-1alpha; TCA, tricarboxylil acid; IL-1β, interleukin-1 beta; PUFAs, long-chain polyunsaturated fatty acids.

There are several molecular pathways involved in the metabolic regulation of the inflammasome: glycolysis, tricarboxylil acid (TCA) cycle, amino-acid metabolism, and fatty acid metabolism, and and most of them have been found to be dysregulated in SSc, providing a potential mechanism involved in inflammasome activation, and thus IL-1β release (52) (**Figure 1**).

Enhanced glycolisis is a hallmark of activated macrophages (53–57). Recents studies of SSc patients undergoing positron emission tomography using the glucose analogue tracer ^18^fluorodeoxyglucose revealed both increased glucose uptake (58). Glycolysis is critical in fibroblast differentiation and has been associated with the development of pulmonary fibrosis in bleomycin-induced experimental models (52). A recent study indicated that TGF‐β1, a key cytokine in scleroderma, up‐regulates glycolysis in dermal fibroblasts derived from SSc patients, and inhibition of glycolysis attenuates its pro‐fibrotic effects (59).

Glutaminolysis through the TCA cycle and its intermediate metabolites was also evaluated in fibrotic conditions. TCA intermediate succinate binds the G-protein-coupled receptor-91 (GPR91) and increases GPR91, type-I collagen, α-SMA, and TGF-β levels. Levels of succinate are up-regulated in lung myofibroblasts of patients with idiopathic pulmonary fibrosis, where they induce TGF-β1, hypoxia-inducible factor-1alpha (HIF-1a), and fibroblast differentiation (58). Of note, succinate levels stabilize HIF-1a and promote IL-1β expression (53); this process is inhibited by itaconate, an anti-inflammatory metabolite required for the activation of the anti-inflammatory transcription factor Nrf2 by lipopolysaccharide in mouse and human macrophages, thus enabling *Nrf2* to increase the expression of downstream anti-oxidant genes as NAD(P)H Quinone Dehydrogenase 1 (60, 61). Interestingly though, the *Nrf2* pathway is highly down-regulated in human and SSc mice with detrimental consequences on inflammation and fibrosis. The *nrf2*^−/−^ mice, indeed, develop a more severe SSc with enhanced fibrosis and inflammation compared to wild-type mice (62).

Results from the aformentioned study about the role of metabolic reprogramming in SSc pathogenesis (59), demonstrated that TGF‐β1 is able to enhance succinate production, which determines an increase of collagen expression, thus providing a link between the pro-fibrotic milieu of the disease and the metabolic activation of the inflammasome. Consistently, SSc fibroblasts incubated with itaconate exhibited reduced expression of collagen (59).

The same study showed that inhibition of glutamine metabolism, another pivotal metabolic pathway fuelling cellular growth, inflammation, and myofibroblast differentiation, antagonises TGF‐β1-induced glycolysis and fibrosis in normal human dermal fibroblasts. Furthermore, SSc fibroblasts showed an increase in glutaminase expression, suggesting that an altered glutamine metabolism may be a hallmark metabolic feature in SSc (59). Also of note, the same glutammate-glutamine pathway has been shown to activate the NLPR3 inflammasome (63).

Finally, fatty acid metabolism might also be implicated in the regulation of NLRP3 inflammasome. However, current evidence is conflicting and synthesis and degradation of fatty acids were linked to inflammasome activation in different studies, perhaps indicating that imbalance itself may activate an inflammatory response. It is also possible that these metabolic pathways activate a common intermediate mediator able to directly activate the NLRP3 inflammasome, the main candidate being mitochondrial reactive oxygen species (mtROS) (53). The ω-3 PUFA, docosahexaenoic acid (DHA), inhibits the activation of caspase-1, thus lowering the production of active IL-1β (64, 65). Apart from DHA, other ω-3 PUFAs, such as eicosapentaenoic acid and α-linolenic acid, can inhibit the activation of the inflammasome (64, 65).

The notion the fatty acid metabolism is dysregulated in SSc dates back to the 1970s, by studies showing that intradermal adipose tissue is progressively replaced by fibrotic tissue in SSc (52, 66).

Taken together, these findings support the existence of a complex biologic loop in SSc, in which the TGF-β1 rich microenvironment, the up-regulated glutamine metabolism, the ehnahced glycolysis, and the fatty acid dysregulation, could all contribute to both inflammasome activation with IL-1β release and myofibroblasts differentiation, thus possibly foraging the occurrence of inflammation-driven fibrosis.

## IL-1 and Heart Inflammation

Recent clinical and experimental data support the relevance of IL-1 in heart inflammation and cardiac dysfunction in several heart diseases. The heart exhibits a highly conserved response to tissue damage, characterized by a stereotyped inflammatory reaction that is centrally mediated by the pro-inflammatory cytokine IL-1 (24). Specifically, IL-1α is released from the dying myocardial cells together with and other intracellular contents, which act as mediators activating the inflammasomes in bystander cells (29, 32, 33, 67–69). IL-1-mediated inflammation ensues; if protracted, this leads to the apoptosis of cardiomyocytes and to the loss of contractile tissue progressively replaced by fibrosis, clinically manifested with cardiomyopathy, HF, and arrhythmic outburst (24).

Previous studies evaluating endomyocardial biopsy (EMB) samples from patients with acute lymphocytic myocarditis indicated that intracellular aggregates of either Apoptosis-associated speck-like protein containing CARD (ASC) or caspase-1, both indicative of inflammasome activation, can cardiomyocytes and infiltrating immune cells. Notably, the number of inflammasome-containing leukocytes correlated with the clinical severity of HF (67).

Moreover, IL-1 causes impaired contractile function by inducing multiple downstream events, including uncoupling of the β-adrenergic receptor from the adenylyl cyclase, inhibition of L-type calcium channels (24, 28, 67–74), transcriptional and post-translational changes in phospholamban and sarcoplasmic/endoplasmic reticulum calcium ATPase (24, 75), mytochondrial dysfunction, and nitric oxide (NO) synthesis (24, 76–78). Animal studies also confirm a role of IL-1-mediated inflammation in HF: injection of plasma from HF patients induced contractile dysfunction in mice, suggesting the existence of cardiodepressant factors in the circulation (79–82). Notably, administration of IL-1β to mice had similar effects, whereas pre-administration of IL-1 inhibitors prevented contractile dysfunction induced by HF serum: collectively considered, these findings indicate that cardiodepressant effects are centrally mediated by IL-1 (75–77), as also in sepsis (82).

Robust evidence also indicates that IL-1 signaling is central to the development of inflammation in both viral and autoimmune acute myocarditis (AMy). Mouse models of coxsackievirus-induced myocarditis exhibit heart infiltration with myeloid cells secreting IL-1 and TNF-α (83). Increased IL-1β expression is also a feature of chronic heart inflammation in experimental models of post-myocarditis DCM, induced by infection with encephalomyocarditis virus (84). Mice lacking IL-1RI did not develop AMy (85), and administration or over-expression of IL-1Ra reduced disease severity in experimental models of cardiomyopathy (86–89). These pre-clinical findings were paralleled by clinical observations in humans: EMBs from patients with viral myocarditis (85) and idiopathic DCM (86) revealed increased IL-1β mRNA levels.

## Heart Inflammation Downstream IL-1

**Figure 2:**
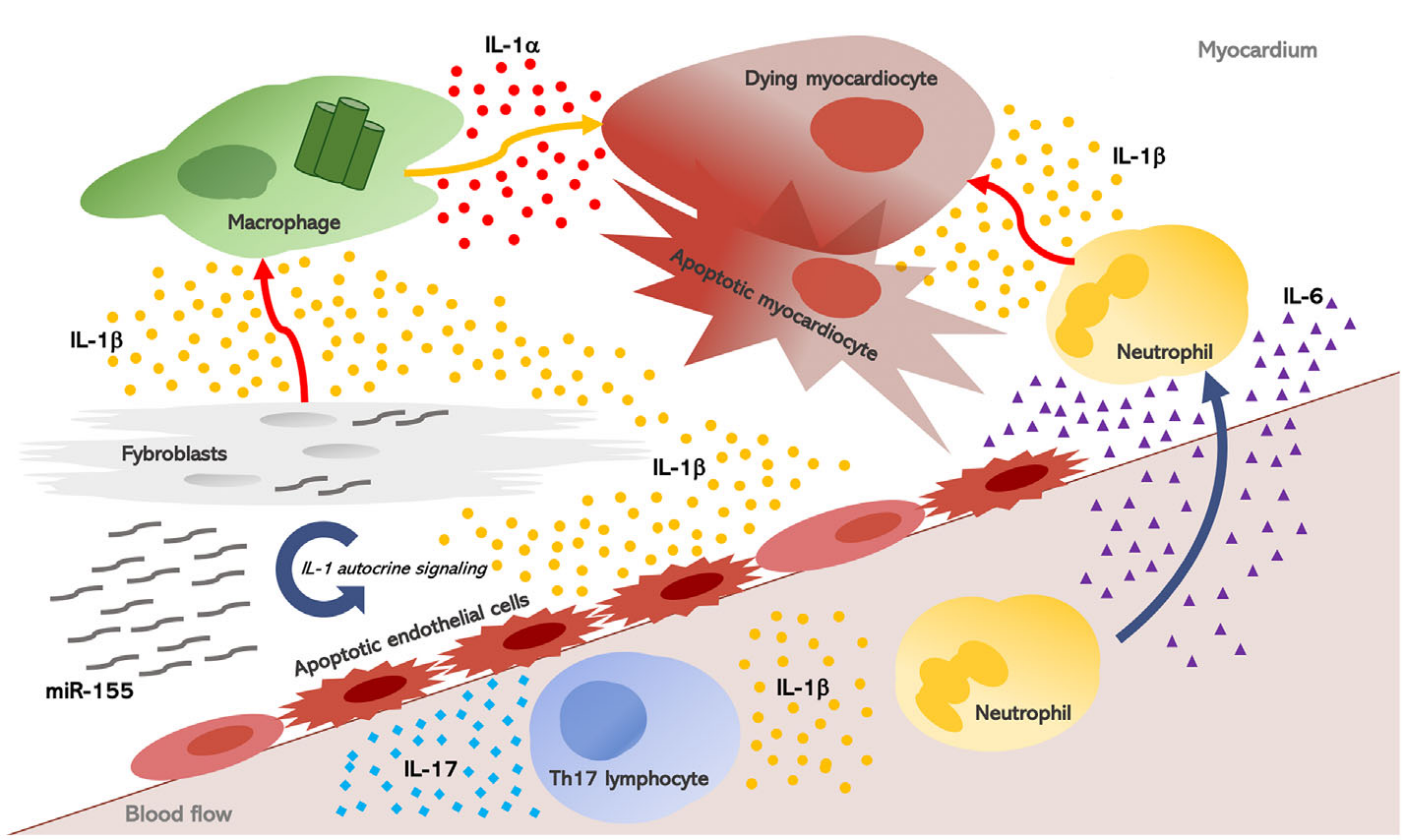
Interleukin-1, myocardial inflammation, and heart fibrosis in systemic sclerosis. Heart inflammation results in myocardial injury. As a consequence, IL-1α is released from dying myocardiocytes, together with intracellular debris and inflammatory mediators; these in turn activate a molecular complex known as the “inflammasome” inside macrophages which processes and releases active IL-1β. Once induced, inflammation escalates into a redundant process: hence, other pro-inflammatory cytokines, mainly IL-6, are produced and they perpetuate heart inflammation and inflammation-driven fibrosis. IL-1 and IL-6 also promote Th17 differentiation, and in post-myocarditis, the role of IL-17A emerged in myocardiac remodeling, thus contributing to both myocardial fibrosis and progression to dilated cardiomyopathy. Finally, in SSc fibroblasts, the NLRP3 inflammasome is over-expressed with consequent increased production of IL-1b. IL-1 also stimulate SSc fibroblasts and induce the synthesis of micro-RNA (miR)-155 which establishes an autocrine loop further increasing IL-1 signaling.

Once induced, inflammation escalates into a redundant process: hence, other pro-inflammatory cytokines may also play a key role in heart inflammation and inflammation-driven fibrosis. The IL-1 biological activity sustains an inflammatory process which involves IL-1 itself as well as downstream mediators. IL-6 is induced by IL-1, and acts as a downstream mediator of several inflammatory effects (**Figure 2**). It is thereby not surprising that IL-6 concentrations are elevated in the serum and myocardium of patients with HF and myocarditis, while also being predictive of adverse outcomes (90). In myocarditis, the primary sources of IL-6 are likely cardiomyocytes and cardiac fibroblasts (91, 92). Overexpression of IL-6 in experimental animals subjected to viral myocarditis results in extensive myocardial inflammation, whereas IL-6 inhibition with tocilizumab reduced heart inflammation and infiltration with CD3+T-cells and CD68+ macrophages (20).

Recently, tocilizumab was used to effectively treat SSc-related myocarditis, and improvement of myocardial inflammation was revealed as a reduction in myocardial edema at cardiac magnetic resonance (CMR), and by the improvement of cardiac function, clinical status and cardiac enzymes (20). Moreover, IL-6 plays a major role in heart fibrosis induced by Ang-II, through TGF-β/Smad activation. Consistently, IL-6 deficiency reduces cardiac inflammation, as well as contractile dysfunction and interstitial fibrosis, without affecting blood pressure in Ang-II-high salt-induced hypertension in IL-6 knockout (IL-6−/−) mice (21). Furthermore, deletion of IL-6 alleviates interstitial fibrosis also in experimental diabetic cardiomyopathy in IL-6−/− mice (81). The deletion or inhibition of miR155 yielded the same protective effects (42, 43), and recent studies revealed that the soluble IL-6 receptor (IL6R) is a target of miR155 (93). In summary, these studies delineate a miR-155/IL-1/IL-6 loop sustaining inflammation-driven fibrosis: overexpression of miR-155 in SSc fibroblasts induces inflammasome-mediated release of IL-1β, which in turn stimulates IL-6 production and collagen synthesis during fibrosis.

Another important signaling axis which potentially contributes to fibrosis and inflammation in SSc is the IL-1/IL-17 axis. Many of inflammatory cytokines that are involved in the SSc pathogenesis, (i.e., IL-1, IL-6, TGF-β), also promote Th17 differentiation. This strongly suggests their potential role in skewing CD4+ T cells toward Th17 differentiation in SSc. A recent *in vivo* study showed that IL-17 is involved in fibrosis and inflammation in bleomycin (BLM)-induced SSc. The authors also used another murine model of SSc, chronic graft-*versus*-host disease(cGVHD), to show that blocking IL-17 activity was able to attenuate disease severity. IL-1 and IL-17 synergically induce the expression of profibrotic and inflammatory mediators, both in human and murine dermal fibroblasts. Subsequent animal studies *in vivo* confirmed the antifibrotic and anti-inflammatory potential of IL-1Ra (94). Hence, IL-17 inhibition, either directly or by blocking IL-1, has therapeutic rationale for tissue fibrosis in SSc. In post-myocarditis, the role of IL-17A emerged either in myocardiac remodeling and the progression to DCM, thus contributing to myocardial fibrosis following experimental AMy by a protein-kinase-C(PKC)b/Erk1/2 Nuclear Factor (NF)-kB signaling (95, 96).

## Therapeutic Applicability of IL-1 Inhibition and Future Perspectives

Despite extensive experimental evidence pointing at a central role for IL-1 in the pathogenesis of heart inflammation, systolic dysfunction, and fibrosis, and despite a possible role of this cytokine in SSc skin and lung inflammation, the use of available IL-1 blocking agents in SSc was only anedoctally reported.

Rilonacept, a fusion protein consisting of the human IL-1 receptor (IL-1R1) and IL-1 receptor accessory protein (IL-1RAcP) which binds and neutralizes both IL-1α and -β, was evaluated in single phase I/II randomized, double-blind, placebo-controlled trial on SSc patients. The primary endpoint was the level of skin expression of the 2G SSc gene biomarkers, which functions as a proxy for the mRSS, while the secondary endpoint was the change in mRSS. Nineteen patients were randomized 2:1 rilonacept 320 mg loading dose at day 0 and then 160 mg weekly *versus* placebo. Skin biopsies were obtained before rilonacept treatment initiation and at week 7. Both the primary and the secondary endpoints were not met, as no changes in gene expression or in the mRSS between treated and placebo patients were observed after 6 weeks (97). However, this trial had several limitations, including the small sample size and the short duration of therapy, even more important in the context of a chronic fibrotic disease. Moreover, no exploratory secondary endpoints to evaluate SSc-HI were considered.

Data from animal models are scarce and conflicting. Treatment with anakinra, a recombinant IL-1 receptor antagonist, improved BLM-induced pulmonary fibrosis in mice and of pulmonary silicosis in humans (98, 99), as well as pulmonary function in patients with COVID-19 and systemic hyperinflammation (100). However, anakinra aggravated pulmonary fibrosis due to Th2 skewing in the fos-related antigen-2 (Fra-2) mouse model of SSc, and was not associated to changes in lung inflammation profile in wild type mice (101). To date, the Fra-2 transgenic mice spontaneously develop pulmonary inflammation. These findings on animal models suggest that the net effect of IL-1 (or of its inhibition) is context-dependent. For this reason, it is still premature to transfer these limited data, obtained on animal models, into clinical practice.

It is important to note that recent evidence supports IL-1 therapeutic blockade in HF and myocarditis. In fact, given the role of IL-1 in both heart inflammation and contractile dysfunction, and the potential role in mechanisms of inflammation-driven heart fibrosis through different pathways, IL-1 inhibition might fit in a proof of concept rationale for an anti-inflammatory therapeutic strategy in SSc myocarditis. In patients with myocardial inflammation and HF, the short-term treatment regimen (14 days) with anakinra improved exercise capability, as determined by oxygen consumption (VO_2_) (79, 102). Prolonged administration (12 weeks) reduced hospitalizations and further improved VO_2_, NTproBNP, and the quality of life in a randomized clinical trial (RCT) (103). Similarly, treatment with the anti–IL-1β monoclonal-antibody canakinumab significantly reduced cardiovascular events in 10,061 patients with previous myocardial infarction and C-reacrive protein >2 mg/L in the CANTOS-trial (104). Recently, our group firstly described the dramatic dampening of heart inflammation and an unprecedented clinical improvement after IL-1 suppression with anakinra in a patient with DCM. Improvement began soon after anakinra administration with improvement of the arrhythmic outburst, decrease of cardiac biomarkers, and normalized echocardiography and CMR imaging—including those that measured LVEF (105). Anakinra treatment was also beneficial in patients with fulminant myocarditis (106–108). Taken together, these observations suggest that IL-1 inhibition could curb heart inflammation while also ameliorating myocardial contractility. Thus, IL-1 inhibition may dampen disease progression and fibrotic damage in patients with myocarditis and other inflammatory cardiomyopathies.

A significant bulk of data also demonstrates the efficacy of IL-1 blockade in pericardial inflammation and recurrent pericarditis (109–113), which usually requires steroids and immunosuppressive treatment when clinically evident (1), including a RCT of anakinra in 21 patients (96), and more recently a positive trial with rilonacept (114). This is of great importance since pericarditis is common in SSc: symptomatic pericarditis occurs in 5–16% patients, whereas autopsy-demonstrated pericardial involvement occurs in 33–72% patients (1).

Beside the aforementioned RCTs in HF (79, 102, 103), double blind, phase IIb, randomized, placebo-controlled clinical trials of anakinra are ongoing to evaluate this treatment in acute myocarditis [ARAMIS-trial, ClinicalTrials.gov: Identifier: NCT03018834] and EMB-proven virus-negative myocarditis [MYTH-1 trial; Eudract: 2018-003472-13]. The monoclonal antibody canakinumab is also clinically available, which blocks IL-1β^111^). More recently, oral NLRP3 inflammasome inhibitors have been proposed to treat a wide spectrum of inflammatory cardiovascular diseases, and RCTs are ongoing (115).

Results from these trials, together with the robust biologic proof of concept, could potentially pave the way to the use of IL-1 therapeutic blockade to treat SSc-related inflammatory heart involvement.

## Data Availability Statement

The original contributions presented in the study are included in the article/supplementary material. Further inquiries can be directed to the corresponding author.

## Author Contributions

GDL: conceived the hypothesis, contributed to the understanding of pathogenic mechanisms of both systemic sclerosis heart involvement and IL-1 mediated heart inflammation, generated original data to support the hypothesis, and drafted the manuscript. GC: contributed to the understanding of biological effects of IL-1 and IL-1 therapeutic blockade in a broad spectrum of rheumatic and inflammatory diseases, generated original data to support the hypothesis, and critically revised the manuscript. CC: contributed to the understanding of pathogenic mechanisms of both systemic sclerosis heart involvement and IL-1 mediated heart inflammation, and critically revised the manuscript. CB: contributed to the understanding of pathogenic mechanisms, clinical presentation, and prognostic meaning of systemic sclerosis, particularly heart involvement, and critically revised the manuscript. AT: contributed to the understanding of biological effects of IL-1 and IL-1 therapeutic blockade in a broad spectrum of rheumatic and inflammatory diseases, created the figures, and critically revised the manuscript. LD: conceived the hypothesis, critically revised the manuscript, and gave the approval of the final version. MM-C: conceived the hypothesis, critically revised the manuscript, and gave the approval of the final version. All authors contributed to the article and approved the submitted version.

## Conflict of Interest

The authors declare that the research was conducted in the absence of any commercial or financial relationships that could be construed as a potential conflict of interest.
